# Elastic Wave Characteristics of Graphene Reinforced Polymer Nanocomposite Curved Beams Including Thickness Stretching Effect

**DOI:** 10.3390/polym12102194

**Published:** 2020-09-25

**Authors:** Pouyan Talebizadehsardari, Arameh Eyvazian, Farayi Musharavati, Roohollah Babaei Mahani, Tamer A. Sebaey

**Affiliations:** 1 Metamaterials for Mechanical, Biomechanical and Multiphysical Applications Research Group, Ton Duc Thang University, Ho Chi Minh City 758307, Vietnam; ptsardari@tdtu.edu.vn; 2 Faculty of Applied Sciences, Ton Duc Thang University, Ho Chi Minh City 758307, Vietnam; 3 Department of Mechanical and Industrial Engineering Qatar University, Qatar University, Doha P.O. Box 2713, Qatar; arameh.eyvazian@gmail.com (A.E.); Musharavati@qu.edu.qa (F.M.); 4 Institute of Research and Development, Duy Tan University, Da Nang 550000, Vietnam; 5 Faculty of Civil Engineering, Duy Tan University, Da Nang 550000, Vietnam; 6 Engineering Management Department, College of Engineering, Prince Sultan University, Riyadh 66833, Saudi Arabia; 7 Mechanical Design and Production Department, Faculty of Engineering, Zagazig University, Sharkia P.O. Box 44519, Egypt

**Keywords:** nanocomposites, graphene nanoplatelets, curved structures, nonlocal strain gradient theory, thickness stretching

## Abstract

This work aims at analyzing elastic wave characteristics in a polymeric nanocomposite curved beam reinforced by graphene nanoplatelets (GNPs). GNPs are adopted as a nanofiller inside the matrix to enhance the effective properties, which are approximated through Halpin-Tasi model and a modified rule of mixture. A higher-order shear deformation theory accounting for thickness stretching and the general strain gradient model to have both nonlocality and strain gradient size-dependency phenomena are adopted to model the nanobeam. A virtual work of Hamilton statement is utilized to get the governing motion equations and is solved in conjunction with the harmonic solution procedure. A comparative study shows the effects of small-scale coefficients, opening angle, weight fraction, the total number of layers in GNPs, and wave numbers on the propagation of waves in reinforced nanocomposite curved beams. This work is also developed for two different distribution of GNPs in a polymeric matrix, namely uniformly distribution and functionally graded one.

## 1. Introduction

Reinforced composite materials comprise a high-strength additive included with the intact resin. Preparing an enhancement in the mechanical characteristics to apply in different industries is the main aim of reinforcing polymeric-metal-ceramic matrixes. Generally, three main ways exist that the reinforcing phase can be incorporated [1]: (a) as material fillers (in the form of individual fillers embedded in the matrix), (b) as grainy material or particulates, and (c) as layers (which are laid on top of one another to make a laminate). The usage of nano-scale derivatives of carbon as a reinforcement phase inside polymer nanocomposites has enhanced electrical, mechanical, and thermal properties [2,3,4,5] (such as stiffness, fatigue and corrosion resistance, and strength-to-weight ratios, and thermal conductivity) of nanostructures because of exclusive characteristics of these nanofillers [6]. Atif et al. [7] reviewed that properties of graphene-based reinforced epoxy nanocomposites in terms of mechanical, thermal, and electrical are associated with the topographical features, weight fraction, morphology, surface functionalization, and dispersion state of graphene. Carbon nanotubes (CNTs), because their stacking sequence caused by wall-to-wall van der Waals interactions, have been considered appropriate candidates for polymer matrix reinforcement. Graphene platelets/nanoplatelets (GPLs/GNPs), which consists of multiple graphene sheets stacked together, have attracted more interest in recent years due to their superior properties. High specific surface areas, high tensile modulus, and lower production cost of GPLS, make them perfect reinforcement material to use in composites which are applicable in the fields of biomedical, aerospace, automotive, and civil engineering [8,9]. GPLs and their polymer composites: fabrication, structure, properties, and applications were reviewed by Shi and his co-authors [10]. Rafee et al. [11] reported that by merging only a 0.1% weight fraction of GPLs inside the reinforced-polymer composite, the mechanical strength, as well as stiffness of the structure, is the same as the degree achieved by merging 1.0% of CNTs. In addition, it was observed that the elastic modulus of the structure amount increases close to 31%. The randomly oriented filler Halpin-Tsai models have been often utilized to predict the material properties of the platelet filler shape in GNP epoxy composites [9]. Afterwards, a great deal of research was carried out on mechanical behaviors of beam/plate/shell structures made of polymeric composites reinforced by graphene nanoplatelets (GNPs) [12,13,14,15,16].

Curved beams, due to their principle curvature plane, have found great importance between useful thin-walled structures, applicable in helicopter, rocket, and vessel type structures. Furthermore, they are joint with thin plate and shell structures to enhance the load capacity in practice. Although a large number of mechanical behaviors on curved beams have been modeled using the Timoshenko beam theory [17] and the Euler-Bernoulli one [18], there are more powerful models obtained by more accurate theories like three-dimensional (3D) theory [19,20]. However, using 3D theory increases the time and effort of the calculation, while higher-order deformation theories [21,22,23] could be considered cost-effective and simpler models for analysis. A sinusoidal beam theory considering the effects of transverse normal stress/strain was developed by Sayyad and Ghugal [24] for finding the bending response of functionally graded (FG) sandwich curved beams. A comprehensive study containing the static bending, buckling, and vibration responses of FG porous graphene-reinforced nanocomposite curved beams was investigated via a trigonometric shear deformation theory by Anirudh et al. [25]. As another point of view, the role of size-dependent effects, which is important in micro/nano-structures, was estimated in the nano-scale curved beam by developing a non-classical higher-order curved beam theory using nonlocal elasticity theory in Ref. [26]. This theory estimates the softening-stiffness mechanism in nanostructures several times [27,28,29]. To point another size-dependent effect, the stiffness-enhancement mechanism [30,31,32,33,34] was examined for the bending behavior of FG curved micro-size beams using the strain gradient theory by Zhang et al. [35]. In another study, Karami et al. [36] examined different micromechanical models to find the size-dependent vibration response of FG curved microbeams using modified coupled stress theory [36]. Recently, the application of the thickness stretching effect was analyzed by developing a new shear deformation theory in Ref. [26]. There are also performed studies in the literature related to mechanical behaviors of curved beams or arches considering the thickness stretching effect and size-dependent mechanisms [37,38,39]. With the advancement in using curved structures inside novel nanostructures [40,41,42], the application of the size-dependent effect in nanocomposite curved beams reinforced by GNPs was analyzed for bending, buckling and vibration responses, in Refs. [43,44], while investigations on other mechanical behaviors are still limited.

One of the practical fields that attracted the attention of researchers in curved beam structures is the propagation of waves [45,46]. Wave propagation is visible in physical phenomena related to geophysics, blood flow, acoustics, non-destructive evaluation, and hydrodynamics. There are additional applications where waves are moving through demanding directions. Moreover, for the scientist who deals with ultrasonic inspection techniques and structural health monitoring, wave propagation response is a determining factor in the design process. Since the non-classical theories can be easily addressed, they have been widely used in size-dependent wave propagation of nanostructures [47,48,49,50]. Wave propagation of FG porous nanobeams based on the nonlocal strain gradient theory was examined by She et al. [51]. Wave dispersion characteristics of nanobeams considering thickness, stretching effect, and size-dependent effects were analyzed by Karami et al. [52]. More recently, the elastic wave characteristic of GNP reinforced composite nanoplates was studied in Reference [53].

The literature reviewed indicates that there is no study covering the elastic wave characteristics of graphene reinforced nanocomposite curved beams, including the thickness and stretching effect. Hence, the presented study is focused on the fourfold coupled (curvilinear axial, rotation, transverse, stretching), size-dependent wave response of a polymeric nanocomposite curved beam reinforced by graphene nanoplatelets (GNPs). The general nonlocal strain gradient model, in order to have both nonlocality and strain gradient size-dependency phenomena are applied to model the curved nanobeam. Hamilton’s principle is applied to derive the governing equations and then they are solved using an analytical plan. Numerical examples show the effects of small-scale coefficients, opening angle, weight fraction as well as the total number of layers in the GNPs, and wave numbers on the propagation of waves in reinforced nanocomposite curved beams.

## 2. Mathematical Formulation

### 2.1. Effective Material Properties for Graphene Reinforced Nanocomposites

**Figure 1:**
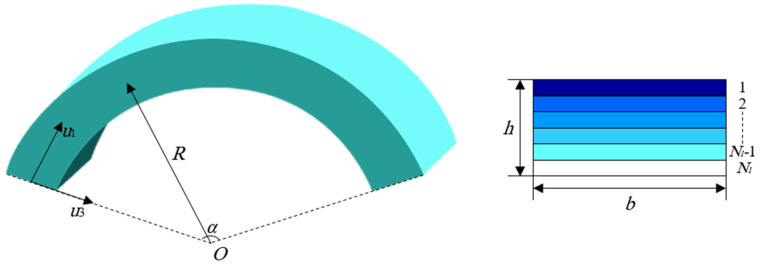
Geometry of graphene nanoplatelets (GNPs) reinforced nanocomposite curved beams.

Figure 1 represents a curved nano-size beam made of polymeric nanocomposites which is reinforced with GNPs consisting of *N_l_* layers with thickness Δ*h* = *h*/*N*_*l*_. The curved beam also includes the length *L*, radius of curvature *R*, opening angle *α*. Here, a modified rule of mixture in conjunction with the Halpin-Tasi model is adopted to get the effective material properties such as Young modulus, density, and Poisson ratio as below [54,55,56]:

(1) $$E_{c}^{\left(k\right)}=\frac{3}{8}\frac{1+\xi_{L}\eta_{L}V_{GNP}^{\left(k\right)}}{1-\eta_{L}V_{GNP}^{\left(k\right)}}\times E_{M}+\frac{5}{8}\frac{1+\xi_{W}\eta_{W}V_{GNP}^{\left(k\right)}}{1-\eta_{W}V_{GNP}^{\left(k\right)}}\times E_{M}$$

(2) $$\eta_{L}=\frac{(E_{GNP}/E_{M})-1}{(E_{GNP}/E_{M})+\xi_{L}}$$

(3) $$\eta_{W}=\frac{(E_{GNP}/E_{M})-1}{(E_{GNP}/E_{M})+\xi_{W}}$$

where *E_M_* and *E_GNP_* are, respectively, Young moduli of the polymer matrix and GNPs; *ξ_L_*, *ξ_W_* indicates the geometrical parameters of GNPs and are given as below:

(4) $$\xi_{L}=2(l_{GNP}/h_{GNP})$$

(5) $$\xi_{W}=2(w_{GNP}/h_{GNP})$$

**Figure 2:**
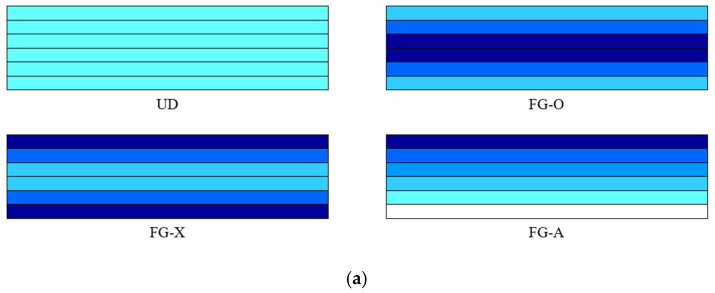

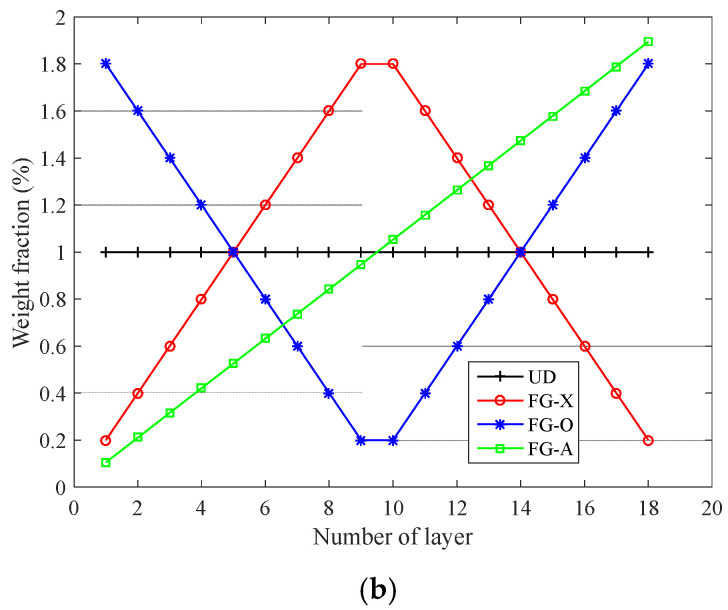
A view of (**a**) the GNPs distribution patterns considering (**b**) its weight fraction variation versus the number of layers.

In the above relations, the average length, width, and thickness of GNPs are, respectively, indicated by *l_GNP_*, *w_GNP_*, and *h_GNP_*. The volume fraction of GNP is defined as follows:

(6) $$V_{GNP}^{\left(k\right)}=\frac{g_{GNP}^{\left(k\right)}}{g_{GNP}^{\left(k\right)}+(\rho_{GNP}/\rho_{M})(1-g_{GNP}^{\left(k\right)})}$$

where the weight fraction of the *k*-layer is showed by *g_GNP_*^(*k*)^; the density of the GNP and polymeric matrix are, respectively, given by *ρ_GNP_* and *ρ_M_*, and the effective density and Poisson ratio are approximated as follows:

(7) $$\rho_{c}^{\left(k\right)}=\rho_{GNP}V_{GNP}^{\left(k\right)}+\rho_{M}V_{GNP}^{\left(k\right)}$$

(8) $$\nu_{c}^{\left(k\right)}=\nu_{GNP}V_{GNP}^{\left(k\right)}+\nu_{M}V_{GNP}^{\left(k\right)}$$

where *ν* is the Poisson ratio. The current work is also expanded for a different distribution of GNPs through the thickness direction (see in Figure 2a) as follows [57]

(9) $$g_{GNP}^{\left(k\right)}=\left\{ \begin{array}{ll} g_{GNP}^{*} & \mathrm{UD}\\ 4g_{GNP}^{*}(\frac{N_{L}+1}{2}-\left|k-\frac{N_{L}+1}{2}\right|)/\left(2+N_{L}\right) & \mathrm{FG}-O\\ 4g_{GNP}^{*}(\frac{1}{2}+\left|k-\frac{N_{L}+1}{2}\right|)/\left(2+N_{L}\right) & \mathrm{FG}-X\\ 2kg_{GNP}^{*}/\left(N_{L}+1\right) & \mathrm{FG}-A \end{array}\right.$$

Furthermore, by using the above relations, the variation of weight fraction in the GNPs for a variety of layers number is illustrated in Figure 2b.

### 2.2. Constitutive Relations

The current work utilized a higher-order shear deformation beam theory that accounts for the thickness stretching effect. The displacement field which has been used in analyzing the different mechanics of beams are given below [26,58,59]:

(10) $$\begin{array}{l} u_{1}=(1+\frac{z}{R})u_{0}(x,t)-z\frac{\partial w_{0}(x,t)}{\partial x}+f(z)\gamma_{0}(x,t)\\ u_{3}=w_{0}(x,t)+zw_{1}(x,t)+z^{2}w_{2}(x,t) \end{array}$$

where $\gamma_{0}(x,t)=\theta (x,t)+\frac{\partial w_{0}(x,t)}{\partial x}-\frac{1}{R}u_{0}(x,t)$; *t* is the time; *u*_0_ and *w_0_* are, respectively, the curvilinear axial displacement and transverse displacement of a mid-line point of the beam; *w*_1,2_ represents the stretching contributions of the displacement in the transverse direction; *θ* is a measure for the rotation of the section. The definition of the function *f*(*z*) is defined as follows:

(11) $$f(z)=\frac{h}{\pi }\sin\frac{\pi z}{h}$$

To obtain the strain components, a curvilinear covariant basis vector is adopted over the displacement field (Equation (10)) as below:

(12) $$\begin{array}{l} \varepsilon_{xx}={\left(1+\frac{z}{R}\right)}^{-1}(\frac{\partial u_{1}}{\partial x}+\frac{u_{3}}{R})\\ \gamma_{xz}=\frac{\partial u_{1}}{\partial z}+{\left(1+\frac{z}{R}\right)}^{-1}(\frac{\partial u_{3}}{\partial x}-\frac{u_{1}}{R})\\ \varepsilon_{zz}=\frac{\partial u_{3}}{\partial z} \end{array}$$

By inserting Equation (10) into the above non-zero strain relation and utilizing some simplifications, the strains are given in the terms of displacements as follows:

(13) $$\begin{array}{l} \varepsilon_{xx}=\frac{\partial u_{0}}{\partial x}+\frac{1}{R}w_{0}+z(\frac{1}{R}\frac{\partial u_{0}}{\partial x}-\frac{\partial^{2}w_{0}}{\partial x^{2}}+\frac{1}{R}w_{1})+\frac{1}{R}z^{2}w_{2}+f(z)\frac{\partial \gamma_{0}}{\partial x}\\ \gamma_{xz}=z\frac{\partial w_{1}}{\partial x}+z^{2}\frac{\partial w_{2}}{\partial x}+\frac{\partial f(z)}{\partial z}\gamma_{0}\\ \varepsilon_{zz}=w_{1}+2zw_{2} \end{array}$$

The equilibrium equations of the curved nanobeam according to a higher-order beam model accounting for fourfold coupled (curvilinear axial, rotation, transverse, stretching) effects can be easily found in the literature, however, they are derived with the help of Hamilton’s principle [26]:

(14) $$\frac{\partial N_{xx}}{\partial x}-\frac{1}{R}(\frac{\partial M_{xx}}{\partial x}-\frac{\partial {\tilde{M}}_{xx}}{\partial x})-\frac{1}{R}\frac{\partial {\tilde{Q}}_{xz}}{\partial x}=-I_{5}\frac{\partial^{2}u_{0}}{\partial t^{2}}-I_{6}\frac{\partial^{3}w_{0}}{\partial x\partial t^{2}}-I_{7}\frac{\partial^{2}\theta }{\partial t^{2}}$$

(15) $$\begin{array}{l} \frac{1}{R}N_{xx}-(\frac{\partial^{2}M_{xx}}{\partial x^{2}}-\frac{\partial^{2}{\tilde{M}}_{xx}}{\partial x^{2}})-\frac{\partial {\tilde{Q}}_{xz}}{\partial x}=I_{6}\frac{\partial^{3}u_{0}}{\partial x\partial t^{2}}+I_{8}\frac{\partial^{4}w_{0}}{\partial x^{2}\partial t^{2}}+I_{9}\frac{\partial^{3}\theta }{\partial x\partial t^{2}}\\ -I_{0}\frac{\partial^{2}w_{0}}{\partial t^{2}}-I_{1}\frac{\partial^{2}w_{1}}{\partial t^{2}}-I_{2}\frac{\partial^{2}w_{2}}{\partial t^{2}} \end{array}$$

(16) $$-\frac{\partial {\tilde{M}}_{xx}}{\partial x}+{\tilde{Q}}_{xz}=-I_{7}\frac{\partial^{2}u_{0}}{\partial t^{2}}-I_{9}\frac{\partial^{3}w_{0}}{\partial x\partial t^{2}}-I_{10}\frac{\partial^{2}\theta }{\partial t^{2}}$$

(17) $$\frac{1}{R}M_{xx}-\frac{\partial Q_{xz}}{\partial x}+N_{zz}=-I_{1}\frac{\partial^{2}w_{0}}{\partial t^{2}}-I_{2}\frac{\partial^{2}w_{1}}{\partial t^{2}}-I_{3}\frac{\partial^{2}w_{2}}{\partial t^{2}}$$

(18) $$\frac{1}{R}{\bar{M}}_{xx}-\frac{\partial {\bar{Q}}_{xz}}{\partial x}+2M_{zz}=-I_{2}\frac{\partial^{2}w_{0}}{\partial t^{2}}-I_{3}\frac{\partial^{2}w_{1}}{\partial t^{2}}-I_{4}\frac{\partial^{2}w_{2}}{\partial t^{2}}$$

where

(19) $$\begin{array}{l} \left[I_{0},I_{1},I_{2},I_{3},I_{4}\right]=\sum_{k=1}^{Nl}\int_{z_{k}}^{z_{+1}}(1,z,z^{2},z^{3},z^{4})\rho_{c}^{\left(k\right)}dz\\ \left[I_{5},I_{6},I_{7}\right]=\sum_{k=1}^{Nl}\int_{z_{k}}^{z_{+1}}(A{\left(z\right)}^{2},A(z)(f(z)-z),A(z)f(z))\rho_{c}^{\left(k\right)}dz\;\mathrm{with}\;A(z)=(1+\frac{z}{R}-\frac{f(z)}{R})\\ \left[I_{8},I_{9},I_{10}\right]=\sum_{k=1}^{Nl}\int_{z_{k}}^{z_{+1}}({\left(f(z)-z\right)}^{2},(f(z)-z)f(z),f{\left(z\right)}^{2})\rho_{c}^{\left(k\right)}dz \end{array}$$

The in-plane normal, shear and bending moments are:

(20) $$\begin{array}{l} \left[N_{xx},M_{xx},{\bar{M}}_{xx},{\tilde{M}}_{xx}\right]=\sum_{k=1}^{Nl}\int_{z_{k}}^{z_{+1}}(1,z,z^{2},f(z))\sigma_{xx}dz\\ \left[N_{zz},M_{zz}\right]=\sum_{k=1}^{Nl}\int_{z_{k}}^{z_{+1}}(1,z)\sigma_{zz}dz\\ \left[Q_{xz},{\bar{Q}}_{xz},{\tilde{Q}}_{xz}\right]=\sum_{k=1}^{Nl}\int_{z_{k}}^{z_{+1}}(z,z^{2},\frac{\partial f(z)}{\partial z})\tau_{xz}dz \end{array}$$

In the current work, the general nonlocal strain gradient theory proposed by Askes and Aifantis [60] is adopted to capture both the nonlocality and strain gradient size dependency. According to this theory, the constitutive size-dependent equations of a curved nanobeam may be defined as:

(21) $$(1-\mu^{2}\frac{\partial^{2}}{\partial x^{2}})\left\{\begin{array}{l} \sigma_{xx}\\ \sigma_{zz}\\ \tau_{xz} \end{array}\right\}=(1-\ell^{2}\frac{\partial^{2}}{\partial x^{2}})\left[\begin{array}{ccc} {\bar{Q}}_{11} & {\bar{Q}}_{13} & 0\\ {\bar{Q}}_{31} & {\bar{Q}}_{33} & 0\\ 0 & 0 & {\bar{Q}}_{55} \end{array}\right]\left\{\begin{array}{l} \varepsilon_{xx}\\ \varepsilon_{zz}\\ \gamma_{xz} \end{array}\right\}$$

where

(22) $$\begin{array}{l} {\bar{Q}}_{11}^{\left(k\right)}={\bar{Q}}_{33}^{\left(k\right)}=\frac{E_{c}^{\left(k\right)}}{1-{\left(\nu_{c}^{\left(k\right)}\right)}^{2}}\\ {\bar{Q}}_{13}^{\left(k\right)}={\bar{Q}}_{31}^{\left(k\right)}=\frac{\nu_{c}^{\left(k\right)}E_{c}^{\left(k\right)}}{1-{\left(\nu_{c}^{\left(k\right)}\right)}^{2}}\\ {\bar{Q}}_{55}^{\left(k\right)}=\frac{E_{c}^{\left(k\right)}}{2+2\nu_{c}^{\left(k\right)}} \end{array}$$

*μ* = (*e*_0_*a*) accounts for nonlocality and is called a nonlocal parameter; *ℓ* represents the strain gradient parameter and captures the stiffness hardening mechanism of nanostructures. By replacing Equation (21) in the resultants presented in Equation (20), it gives the following resultants:

(23) $$\begin{array}{l} \left(1-\mu^{2}\frac{\partial^{2}}{\partial x^{2}})N_{xx}=(1-\ell^{2}\frac{\partial^{2}}{\partial x^{2}})(A_{11}(\frac{\partial u_{0}}{\partial x}+\frac{1}{R}w_{0})+B_{11}(\frac{1}{R}\frac{\partial u_{0}}{\partial x}-\frac{\partial^{2}w_{0}}{\partial x^{2}}+\frac{1}{R}w_{1}\right)\\ +D_{11}\frac{1}{R}w_{2}+E_{11}\frac{\partial \gamma_{0}}{\partial x}+A_{13}w_{1}+2B_{13}w_{2}) \end{array}$$

(24) $$\begin{array}{l} \left(1-\mu^{2}\frac{\partial^{2}}{\partial x^{2}})M_{xx}=(1-\ell^{2}\frac{\partial^{2}}{\partial x^{2}})(B_{11}(\frac{\partial u_{0}}{\partial x}+\frac{1}{R}w_{0})+D_{11}(\frac{1}{R}\frac{\partial u_{0}}{\partial x}-\frac{\partial^{2}w_{0}}{\partial x^{2}}+\frac{1}{R}w_{1}\right)\\ +F_{11}\frac{1}{R}w_{2}+G_{11}\frac{\partial \gamma_{0}}{\partial x}+B_{13}w_{1}+2D_{13}w_{2}) \end{array}$$

(25) $$\begin{array}{l} \left(1-\mu^{2}\frac{\partial^{2}}{\partial x^{2}}){\bar{M}}_{xx}=(1-\ell^{2}\frac{\partial^{2}}{\partial x^{2}})(D_{11}(\frac{\partial u_{0}}{\partial x}+\frac{1}{R}w_{0})+F_{11}(\frac{1}{R}\frac{\partial u_{0}}{\partial x}-\frac{\partial^{2}w_{0}}{\partial x^{2}}+\frac{1}{R}w_{1}\right)\\ +H_{11}\frac{1}{R}w_{2}+J_{11}\frac{\partial \gamma_{0}}{\partial x}+D_{13}w_{1}+2F_{13}w_{2}) \end{array}$$

(26) $$\begin{array}{l} \left(1-\mu^{2}\frac{\partial^{2}}{\partial x^{2}}){\tilde{M}}_{xx}=(1-\ell^{2}\frac{\partial^{2}}{\partial x^{2}})(D_{11}(\frac{\partial u_{0}}{\partial x}+\frac{1}{R}w_{0})+G_{11}(\frac{1}{R}\frac{\partial u_{0}}{\partial x}-\frac{\partial^{2}w_{0}}{\partial x^{2}}+\frac{1}{R}w_{1}\right)\\ +J_{11}\frac{1}{R}w_{2}+K_{11}\frac{\partial \gamma_{0}}{\partial x}+G_{13}w_{1}+2J_{13}w_{2}) \end{array}$$

(27) $$\begin{array}{l} \left(1-\mu^{2}\frac{\partial^{2}}{\partial x^{2}})N_{zz}=(1-\ell^{2}\frac{\partial^{2}}{\partial x^{2}})(A_{31}(\frac{\partial u_{0}}{\partial x}+\frac{1}{R}w_{0})+B_{31}(\frac{1}{R}\frac{\partial u_{0}}{\partial x}-\frac{\partial^{2}w_{0}}{\partial x^{2}}+\frac{1}{R}w_{1}\right)\\ +D_{31}\frac{1}{R}w_{2}+E_{31}\frac{\partial \gamma_{0}}{\partial x}+A_{33}w_{1}+2B_{33}w_{2}) \end{array}$$

(28) $$\begin{array}{l} \left(1-\mu^{2}\frac{\partial^{2}}{\partial x^{2}})M_{zz}=(1-\ell^{2}\frac{\partial^{2}}{\partial x^{2}})(B_{31}(\frac{\partial u_{0}}{\partial x}+\frac{1}{R}w_{0})+D_{31}(\frac{1}{R}\frac{\partial u_{0}}{\partial x}-\frac{\partial^{2}w_{0}}{\partial x^{2}}+\frac{1}{R}w_{1}\right)\\ +F_{31}\frac{1}{R}w_{2}+G_{31}\frac{\partial \gamma_{0}}{\partial x}+B_{33}w_{1}+2D_{33}w_{2}) \end{array}$$

(29) $$\left(1-\mu^{2}\frac{\partial^{2}}{\partial x^{2}})Q_{xz}=(1-\ell^{2}\frac{\partial^{2}}{\partial x^{2}})(D_{55}\frac{\partial w_{1}}{\partial x}+F_{55}\frac{\partial w_{2}}{\partial x}+L_{55}\gamma_{0}\right)$$

(30) $$\left(1-\mu^{2}\frac{\partial^{2}}{\partial x^{2}}){\bar{Q}}_{xz}=(1-\ell^{2}\frac{\partial^{2}}{\partial x^{2}})(F_{55}\frac{\partial w_{1}}{\partial x}+H_{55}\frac{\partial w_{2}}{\partial x}+M_{55}\gamma_{0}\right)$$

(31) $$\left(1-\mu^{2}\frac{\partial^{2}}{\partial x^{2}}){\tilde{Q}}_{xz}=(1-\ell^{2}\frac{\partial^{2}}{\partial x^{2}})(L_{55}\frac{\partial w_{1}}{\partial x}+M_{55}\frac{\partial w_{2}}{\partial x}+N_{55}\gamma_{0}\right)$$

where

(32) $$\left[\begin{array}{cccc} A_{ij} & B_{ij} & D_{ij} & E_{ij}\\ F_{ij} & G_{ij} & H_{ij} & J_{ij}\\ K_{ij} & L_{ij} & M_{ij} & N_{ij} \end{array}\right]=\sum_{k=1}^{Nl}\int_{z_{k}}^{z_{+1}}\left[\begin{array}{cccc} 1 & z & z^{2} & f(z)\\ z^{3} & zf(z) & z^{4} & z^{2}f(z)\\ f{\left(z\right)}^{2} & z\frac{\partial f(z)}{\partial z} & z^{2}\frac{\partial f(z)}{\partial z} & \frac{\partial f(z)}{\partial z}\frac{\partial f(z)}{\partial z} \end{array}\right]Q_{ij}dz$$

Ultimately, the equations of motion in the term of displacements for a polymeric nanocomposite curved beam reinforced with GNPs accounting for both nonlocal and strain gradient coefficients might be derived in the following form by placing Equations (23)–(31), into Equations (14)–(18):

(33) $$\begin{array}{l} \left(1-\ell^{2}\frac{\partial^{2}}{\partial x^{2}})(A_{11}(\frac{\partial^{2}u_{0}}{\partial x^{2}}+\frac{1}{R}\frac{\partial w_{0}}{\partial x})+B_{11}(\frac{1}{R}\frac{\partial^{2}u_{0}}{\partial x^{2}}-\frac{\partial^{3}w_{0}}{\partial x^{3}}+\frac{1}{R}\frac{\partial w_{1}}{\partial x}\right)\\ +D_{11}\frac{1}{R}\frac{\partial w_{2}}{\partial x}+E_{11}\frac{\partial^{2}\gamma_{0}}{\partial x^{2}}+A_{13}\frac{\partial w_{1}}{\partial x}+2B_{13}\frac{\partial w_{2}}{\partial x}-\frac{1}{R}\langle B_{11}(\frac{\partial^{2}u_{0}}{\partial x^{2}}+\frac{1}{R}\frac{\partial w_{0}}{\partial x})\\ +D_{11}(\frac{1}{R}\frac{\partial^{2}u_{0}}{\partial x^{2}}-\frac{\partial^{3}w_{0}}{\partial x^{3}}+\frac{1}{R}\frac{\partial w_{1}}{\partial x})+F_{11}\frac{1}{R}\frac{\partial w_{2}}{\partial x}+G_{11}\frac{\partial^{2}\gamma_{0}}{\partial x^{2}}+B_{13}\frac{\partial w_{1}}{\partial x}+2D_{13}\frac{\partial w_{2}}{\partial x}\\ -D_{11}(\frac{\partial^{2}u_{0}}{\partial x^{2}}+\frac{1}{R}\frac{\partial w_{0}}{\partial x})-G_{11}(\frac{1}{R}\frac{\partial^{2}u_{0}}{\partial x^{2}}-\frac{\partial^{3}w_{0}}{\partial x^{3}}+\frac{1}{R}\frac{\partial w_{1}}{\partial x})-J_{11}\frac{1}{R}\frac{\partial w_{2}}{\partial x}\\ -K_{11}\frac{\partial^{2}\gamma_{0}}{\partial x^{2}}-G_{13}\frac{\partial w_{1}}{\partial x}-2J_{13}\frac{\partial w_{2}}{\partial x}+L_{55}\frac{\partial^{2}w_{1}}{\partial x^{2}}+M_{55}\frac{\partial^{2}w_{2}}{\partial x^{2}}+N_{55}\frac{\partial \gamma_{0}}{\partial x}\rangle \\ =(1-\mu^{2}\frac{\partial^{2}}{\partial x^{2}})(-I_{5}\frac{\partial^{2}u_{0}}{\partial t^{2}}-I_{6}\frac{\partial^{3}w_{0}}{\partial x\partial t^{2}}-I_{7}\frac{\partial^{2}\theta }{\partial t^{2}}) \end{array}$$

(34) $$\begin{array}{l} (1-\ell^{2}\frac{\partial^{2}}{\partial x^{2}})(\frac{1}{R}\langle \left(A_{11}(\frac{\partial u_{0}}{\partial x}+\frac{1}{R}w_{0})+B_{11}(\frac{1}{R}\frac{\partial u_{0}}{\partial x}-\frac{\partial^{2}w_{0}}{\partial x^{2}}+\frac{1}{R}w_{1}\right)+D_{11}\frac{1}{R}w_{2}\\ +E_{11}\frac{\partial \gamma_{0}}{\partial x}+A_{13}w_{1}+2B_{13}w_{2})\rangle -\langle B_{11}(\frac{\partial^{3}u_{0}}{\partial x^{3}}+\frac{1}{R}\frac{\partial^{2}w_{0}}{\partial x^{2}})+D_{11}(\frac{1}{R}\frac{\partial^{3}u_{0}}{\partial x^{3}}-\frac{\partial^{4}w_{0}}{\partial x^{4}}\\ +\frac{1}{R}\frac{\partial^{2}w_{1}}{\partial x^{2}})+F_{11}\frac{1}{R}\frac{\partial^{2}w_{2}}{\partial x^{2}}+G_{11}\frac{\partial^{3}\gamma_{0}}{\partial x^{3}}+B_{13}\frac{\partial^{2}w_{1}}{\partial x^{2}}+2D_{13}\frac{\partial^{2}w_{2}}{\partial x^{2}}\rangle +D_{11}(\frac{\partial^{3}u_{0}}{\partial x^{3}}+\frac{1}{R}\frac{\partial^{2}w_{0}}{\partial x^{2}})\\ +G_{11}(\frac{1}{R}\frac{\partial^{3}u_{0}}{\partial x^{3}}-\frac{\partial^{4}w_{0}}{\partial x^{4}}+\frac{1}{R}\frac{\partial^{2}w_{1}}{\partial x^{2}})+J_{11}\frac{1}{R}\frac{\partial^{2}w_{2}}{\partial x^{2}}+K_{11}\frac{\partial^{3}\gamma_{0}}{\partial x^{3}}+G_{13}\frac{\partial^{2}w_{1}}{\partial x^{2}}+2J_{13}\frac{\partial^{2}w_{2}}{\partial x^{2}}\\ -\langle L_{55}\frac{\partial^{2}w_{1}}{\partial x^{2}}+M_{55}\frac{\partial^{2}w_{2}}{\partial x^{2}}+N_{55}\frac{\partial \gamma_{0}}{\partial x}\rangle )=(1-\mu^{2}\frac{\partial^{2}}{\partial x^{2}})(I_{6}\frac{\partial^{3}u_{0}}{\partial x\partial t^{2}}+I_{8}\frac{\partial^{4}w_{0}}{\partial x^{2}\partial t^{2}}+I_{9}\frac{\partial^{3}\theta }{\partial x\partial t^{2}}\\ -I_{0}\frac{\partial^{2}w_{0}}{\partial t^{2}}-I_{1}\frac{\partial^{2}w_{1}}{\partial t^{2}}-I_{2}\frac{\partial^{2}w_{2}}{\partial t^{2}}) \end{array}$$

(35) $$\begin{array}{l} (1-\ell^{2}\frac{\partial^{2}}{\partial x^{2}})(-\langle \left(D_{11}(\frac{\partial u_{0}}{\partial x}+\frac{1}{R}w_{0})+G_{11}(\frac{1}{R}\frac{\partial u_{0}}{\partial x}-\frac{\partial^{2}w_{0}}{\partial x^{2}}+\frac{1}{R}w_{1}\right)\\ +J_{11}\frac{1}{R}w_{2}+K_{11}\frac{\partial \gamma_{0}}{\partial x}+G_{13}w_{1}+2J_{13}w_{2})\rangle +L_{55}\frac{\partial w_{1}}{\partial x}+M_{55}\frac{\partial w_{2}}{\partial x}+N_{55}\gamma_{0})\\ =(1-\mu^{2}\frac{\partial^{2}}{\partial x^{2}})(-I_{7}\frac{\partial^{2}u_{0}}{\partial t^{2}}-I_{9}\frac{\partial^{3}w_{0}}{\partial x\partial t^{2}}-I_{10}\frac{\partial^{2}\theta }{\partial t^{2}}) \end{array}$$

(36) $$\begin{array}{l} (1-\ell^{2}\frac{\partial^{2}}{\partial x^{2}})(\frac{1}{R}\langle B_{11}(\frac{\partial u_{0}}{\partial x}+\frac{1}{R}w_{0})+D_{11}(\frac{1}{R}\frac{\partial u_{0}}{\partial x}-\frac{\partial^{2}w_{0}}{\partial x^{2}}+\frac{1}{R}w_{1})\\ +F_{11}\frac{1}{R}w_{2}+G_{11}\frac{\partial \gamma_{0}}{\partial x}+B_{13}w_{1}+2D_{13}w_{2})\rangle -\langle D_{55}\frac{\partial^{2}w_{1}}{\partial x^{2}}+F_{55}\frac{\partial^{2}w_{2}}{\partial x^{2}}+L_{55}\frac{\partial \gamma_{0}}{\partial x}\rangle \\ +A_{31}(\frac{\partial u_{0}}{\partial x}+\frac{1}{R}w_{0})+B_{31}(\frac{1}{R}\frac{\partial u_{0}}{\partial x}-\frac{\partial^{2}w_{0}}{\partial x^{2}}+\frac{1}{R}w_{1})+D_{31}\frac{1}{R}w_{2}+E_{31}\frac{\partial \gamma_{0}}{\partial x}\\ +A_{33}w_{1}+2B_{33}w_{2})=(1-\mu^{2}\frac{\partial^{2}}{\partial x^{2}})(-I_{1}\frac{\partial^{2}w_{0}}{\partial t^{2}}-I_{2}\frac{\partial^{2}w_{1}}{\partial t^{2}}-I_{3}\frac{\partial^{2}w_{2}}{\partial t^{2}}) \end{array}$$

(37) $$\begin{array}{l} (1-\ell^{2}\frac{\partial^{2}}{\partial x^{2}})(\frac{1}{R}\langle D_{11}(\frac{\partial u_{0}}{\partial x}+\frac{1}{R}w_{0})+F_{11}(\frac{1}{R}\frac{\partial u_{0}}{\partial x}-\frac{\partial^{2}w_{0}}{\partial x^{2}}+\frac{1}{R}w_{1})\\ +H_{11}\frac{1}{R}w_{2}+J_{11}\frac{\partial \gamma_{0}}{\partial x}+D_{13}w_{1}+2F_{13}w_{2}\rangle -\langle F_{55}\frac{\partial^{2}w_{1}}{\partial x^{2}}+H_{55}\frac{\partial^{2}w_{2}}{\partial x^{2}}+M_{55}\frac{\partial \gamma_{0}}{\partial x}\rangle \\ +2\langle B_{31}(\frac{\partial u_{0}}{\partial x}+\frac{1}{R}w_{0})+D_{31}(\frac{1}{R}\frac{\partial u_{0}}{\partial x}-\frac{\partial^{2}w_{0}}{\partial x^{2}}+\frac{1}{R}w_{1})+B_{33}w_{1}+2D_{33}w_{2}\\ +F_{31}\frac{1}{R}w_{2}+G_{31}\frac{\partial \gamma_{0}}{\partial x}\rangle )=(1-\mu^{2}\frac{\partial^{2}}{\partial x^{2}})(-I_{2}\frac{\partial^{2}w_{0}}{\partial t^{2}}-I_{3}\frac{\partial^{2}w_{1}}{\partial t^{2}}-I_{4}\frac{\partial^{2}w_{2}}{\partial t^{2}}) \end{array}$$

## 3. Solution Procedure

Light waves and mechanical waves are two branches of elastic waves. The current work supports mechanical waves that happen by oscillations of matter. Furthermore, the present work studied bulk waves which are far from boundaries. In this section, a series of harmonics is adopted to solve the formulation to obtain the appropriate system response as shown below:

(38) $$\begin{array}{l} u_{0}=U\exp(ix\beta -i\omega t)\\ \theta =\Theta \exp(ix\beta -i\omega t)\\ w_{0}=W_{0}\exp(ix\beta -i\omega t)\\ w_{1}=W_{1}\exp(ix\beta -i\omega t)\\ w_{2}=W_{2}\exp(ix\beta -i\omega t) \end{array}$$

In which ($U,\Theta ,W_{0},W_{1},W_{2}$) denote amplitudes; *β* is the wave number; *ω* represents the wave frequency. To get the dynamic response of the system, we used a standard eigenvalue problem which is observable through substituting Equation (38) into Equations (33)–(37) as below,

(39) $$({\left[K\right]}_{5\times 5}-\omega^{2}{\left[M\right]}_{5\times 5})\left\{X\right\}=0$$

Herein [**K**] and [**M**] indicate, respectively, the stiffness and mass matrixes; and the eigenvector can be given by $\left\{X\right\}={\left\{U,\Theta ,W_{0},W_{1},W_{2}\right\}}^{T}$. The phase velocity can be found by *c = ω/β*.

## 4. Numerical Examples

The current study deals with an investigation on the propagated wave in a curved nanobeam made of GNPs reinforced polymeric nanocomposite considering size effects and the thickness stretching effect for the first time. Higher-order shear deformation beam theory is utilized to model the curved beam as a continuum model where the nonlocal strain gradient theory (NSGT) is utilized to estimate the size-dependent effects. The obtained governing equations through the Hamiltonian principle are solved via an analytical solution.

### 4.1. Comparison Study

Firstly, in order to validate the proposed size-dependent model, the eigenvalue natural frequency of the isotropic curved nanobeam is obtained and compared with those derived by other theories [26], and tabulated in Table 1, in which the assumption of nonlocal elasticity is also considered. The natural frequencies are presented for different opening angles and nonlocal parameters for a constant thickness ratio L/h = 10. Comparing the current results with those reported in Ref. [26], shows an adequate level of agreement. It is interesting to note that when the opening angle tends to infinity (increases), the curved nanobeam behaves like a straight one.

### 4.2. Parametric Studies

A polymeric matrix with Young’s modulus *E_M_* = 3 GPa, density *ρ_M_* = 1200 kg/m^3^, and Poisson’s ratio *ν_M_* = 0.34 is modelled in this work; for the reinforcement process, the GNP is used supporting the Young modulus *E_GNP_* = 1.01 TPa, density *ρ_GNP_* = 1060 kg/m^3^ and Poisson’s ratio *ν_GNP_* = 0.186. The whole thickness of the selected polymeric nanocomposite curved beam is h = 20 nm, with in-plane geometry of *a* = *b*=200 nm, which is made of GNPs with length *l_GNP_* = 3 nm, thickness *h_GNP_* = 7 nm, and width *w_GNP_* = 1.8 nm [61]. Moreover, the number of involved layers is considered 10, except when it is said.

**Figure 3:**
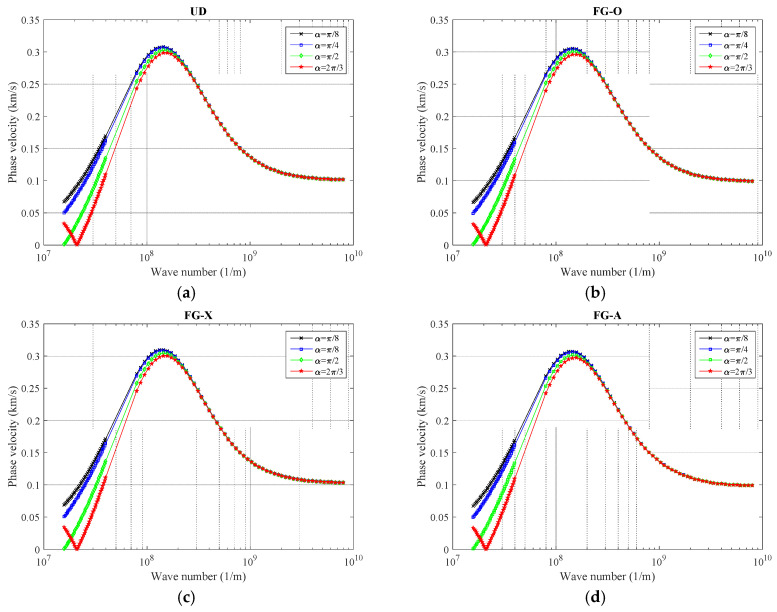
The variation of phase velocity versus opening angle for (**a**) uniform distribution (UD), (**b**) FG-O, (**c**) FG-X, and (**d**) FG-A patterns of GNPs, (*ℓ* = 1 nm, *μ* = 10 nm, g^*^_GNP_ = 1%, *N_l_* = 10).

As discussed before, there are different patterns to model the GNPs inside polymer-reinforced nanocomposites. In Figure 3, the phase velocity of graphene reinforced polymeric nanocomposite curved beams made of various GNPs distribution schemes (UD, FX-O, FG-X, and FG-A) are illustrated for multiple opening angle values. For this purpose, the wave number is flexible for 0.01 to 100 (1/nm). As observed, when the wave number increases, the phase velocity curves follow a similar trend independently of the GNP scheme so that the phase velocity is increased to its peak around 1 × 10^8^ (1/m), and then becomes moderate for high wave numbers after a sharp decline. Regarding the role of the opening angle, as it decreases the phase velocity is increased due to the fact that with a higher angle of curvature, the curved nanobeam converts to a softer structure with lower frequencies. However, there is an exception for *α =* 2*π*/3 when the wave number is supposed to be very low (1 × 10^8^ (1/m)). To be more precise, the opening angle is a more prominent factor at low wave numbers while for high wave numbers, change in the curvature of the curved nanobeam is not an influential factor in phase velocity variations. Moreover, it can be seen that FG-X properties have the highest wave frequencies; the FG-O behaves oppositely, while the gap between wave responses of UD and FG-A patterns seems to be small.

**Figure 4:**
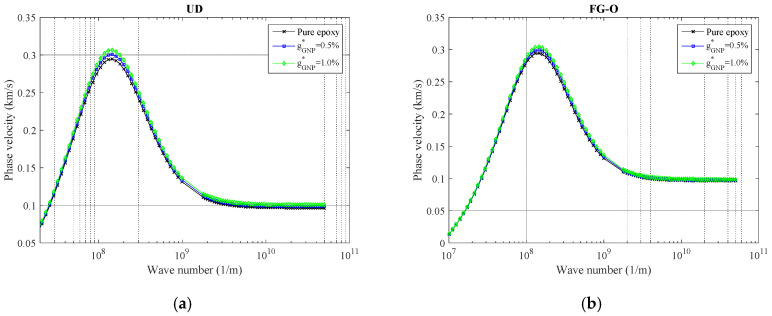

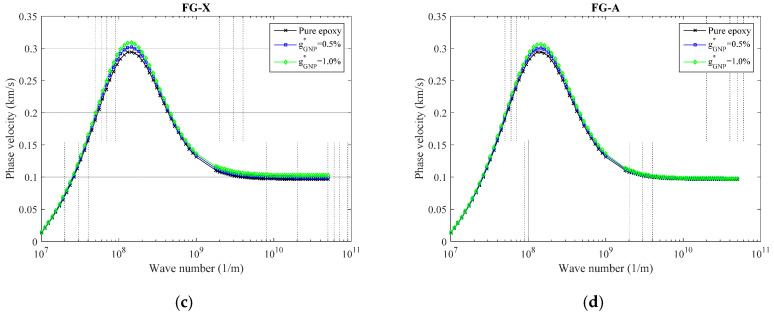
The variation of phase velocity versus weight fraction of GNPs for (**a**) UD, (**b**) FG-O, (**c**) FG-X, and (**d**) FG-A patterns of GNPs (*α* = π/4, *ℓ* = 1 nm, *μ* = 10 nm, *N_l_* = 10).

To investigate the impact of the GNPs weight fraction on wave response for the polymeric nanocomposite curved beam reinforced by the GNPs for different wave numbers, Figure 4 is plotted based on phase velocity responses. Pure epoxy indicates the g^*^_GNP_ = 0, while it increases to 1%. In fact, the higher GNPs weight fraction values create a harder nanocomposite curved beam by raising its stiffness which resulted in higher phase velocities. This phenomenon can be easily observed in peaks and also high wave numbers.

**Figure 5:**
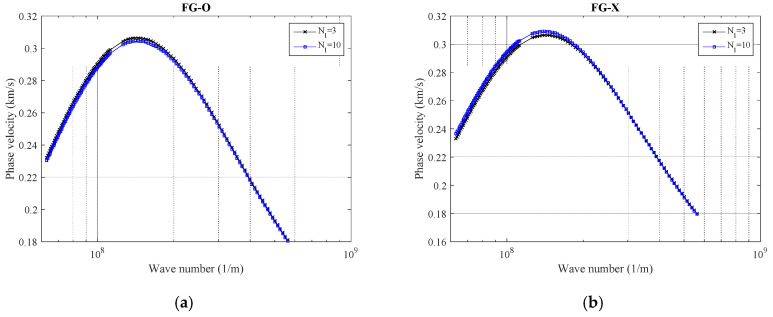

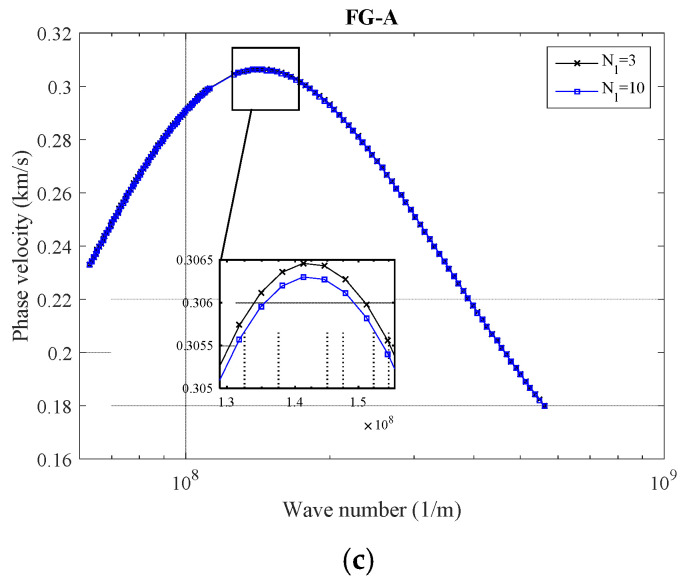
The variation of phase velocity versus number of layers in GNPs for (**a**) FG-O, (**b**) FG-X, and (**c**) FG-A patterns of GNPs (*α* = π/4, *ℓ* = 1 nm, *μ* = 10 nm, g^*^_GNP_ = 1%)**.**

Until now, the total number of involved layers is supposed to be 10. In Figure 5, wave response trends of the nanobeam reinforced by GNP result from different GNP distribution schemes with a variable number of layers plotted. To prepare this model, the total number is driven for 3 and 10 layers of GNPs. For the selected number of layers in this example (i.e., 3 and 10), regardless of the GNP distribution scheme, the reduction in the number of layers can increase somewhat the material capability of reinforced nanocomposite in which an increment in the phase velocity index is a result. However, in order to obtain a more accurate observation, one must be focused on Figure 2b, where the weight fraction index shows in terms of the number of layers. The reason behind that may be related to the internal interactions of nanobeam atoms so that the visibility of this phenomenon is more at peaks, where the maximum results are observed. Furthermore, as seen previously, FG-X provides the lowest phase velocity while the highest ones are resulted from the FG-O model. It is interesting to note that for the FG-A pattern, variation in the number of involved layers seems to have minimal effect on results.

**Figure 6:**
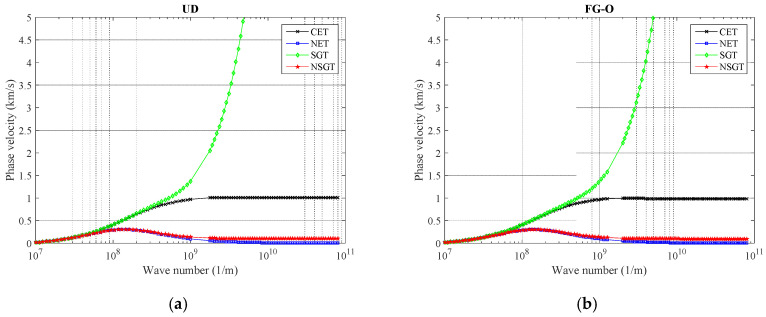

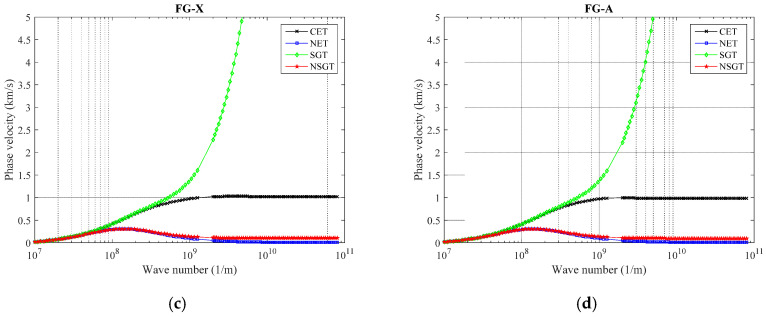
The variation of phase velocity versus different size-dependent theories for (**a**) UD, (**b**) FG-O, (**c**) FG-X, and (**d**) FG-A patterns of GNPs (*α* = π/4, *ℓ* = 1 nm, *μ* = 10 nm, g^*^_GNP_ = 1%, *N_l_* = 10).

The graphical relationships between wave number and phase velocity for the curved nanobeam reinforced by GNPs based on various classical elasticity theory (CET), as well as size-dependent theories including nonlocal elasticity theory (NET), strain gradient theory (SGT), and NSGT are illustrated in Figure 6. As graphed in Figure 6, it can be observed that the phase velocity curves calculated by the CET, NET, SGT, and NSGT are almost identical when the wave number is lower than 0.1 (1/nm). This shows that, for small wave numbers [*k* < 0.1 (1/nm)], phase velocity of small-scale structures (such as a curved nanobeam) is not sensitive to size-dependent effects, and the dependence of phase velocity to size effects (such as softening-stiffness and stiffness-enhancement mechanisms) is more significant for higher wave numbers. In fact, the softening-stiffness mechanism controlled by the nonlocal parameter and stiffness-enhancement effect controlled by the strain gradient parameter respectively cause the decrease and increase in the phase velocity owing to the fact that the curved nanobeam becomes a softer or harder structure, respectively. However, to obtain a more general observation of size-dependent efficiency may require more concentrated results for higher wave numbers of reinforced nanocomposites. It is because as the nonlocal parameter enhances, the GNPs reinforced polymeric nanocomposite curved beam converts to a softer structure, and as a result, the phase velocity is reduced. The aforementioned result is reserved for an increase in the strain gradient parameter.

## 5. Conclusions

The governing equations for size-dependent polymeric nanocomposite curved beam reinforced by GNPs were derived using a higher-order shear deformation beam theory including the stretching effect by following the assumption of nonlocal strain gradient theory, and afterward, wave frequency and phase velocity of the structures were evaluated in this research. An analytical solution was applied to determine the wave propagation response, while a non-classical model estimated softening-stiffness and stiffness-enhancement mechanisms inside the nanostructure. The model compared favorably with the available examples in the open literature.

Based on the parametric studies for the wave propagation of the curved nanobeam reinforced with GNPs performed in this study, the following conclusions are summarized:

- As wave number increases, the phase velocity obtained by non-classical theories is increased until a unique value for the wave number (around 1 × 10^8^ (1/m)), and afterwards, a sharp decrease in phase velocity is a result of an increase in the wave number.
- With the increment in the nonlocal parameter (strain gradient parameter), the phase velocity of the curved nanobeam are decreased (increased).
- In the case of high wave numbers, the role of the opening angle on the wave characteristics is not significant; while at low wave numbers, as the opening angle increases, the results declined.
- To determine the GNP efficiently, the maximum phase velocity belongs to FG-X reinforcement model of polymeric nanocomposite curved beam, followed by UD, FG-A, and FG-O models, independent to the number of layers.
- As number of layers increases, the phase velocity gap between different GNP types is increased.
- Regardless of GNPs scheme, an increment in GNPs weight fraction, the curved nanobeam becomes harder and consequently its phase velocity is decreased.

## Figures and Tables

**Table 1:** A comparison between the natural frequency of isotropic curved nanobeam (*L/h =* 10).

	*α =* π/6		*α =* π/3		*α =* π/2		*α =* 2π/3	
Model	*μ*^2^*=* 0	*μ*^2^*=* 2	*μ*^2^*=* 0	*μ*^2^*=* 2	*μ*^2^*=* 0	*μ*^2^*=* 2	*μ*^2^*=* 0	*μ*^2^*=* 2
CLT [26]	9.4253	8.6134	8.2864	7.5726	6.5913	6.0235	4.5424	4.1511
FSDT [26]	9.3276	8.5242	8.2004	7.4941	6.5228	5.9609	4.4951	4.1079
HSDT [26]	9.3142	8.5119	8.1888	7.4834	6.5131	5.9521	4.4873	4.1008
Present (*ε_z_ ≠* 0)	9.31419	8.81191	8.18879	7.48344	6.51307	5.95206	4.48727	4.10076
